# Excessive GABAergic activity in striatal and frontal cortical regions more than dopaminergic functions are related to daytime sleepiness in Parkinson’s disease: An exploratory 11C-flumazenil PET study

**DOI:** 10.70401/geromedicine.2026.0025

**Published:** 2026-05-20

**Authors:** Robert Vangel, Stiven Roytman, Madhusudan Reddy, August Van Hout, Mélanie L. Beaulieu, Giulia Carli, Peter J.H. Scott, Prabesh Kanel, Nicolaas I. Bohnen

**Affiliations:** 1 Department of Radiology, University of Michigan, Ann Arbor, MI 48109, USA.; 2 Department of Neurology, University of Michigan, Ann Arbor, MI 48109, USA.; 3 Morris K. Udall Center of Excellence for Parkinson’s Disease Research, University of Michigan, Ann Arbor, MI 48109, USA.; 4 Neurology Service and GRECC, VA Ann Arbor Healthcare System, Ann Arbor, MI 48105, USA.

**Keywords:** GABA_A_ receptor, Parkinson’s disease, excessive daytime sleepiness, positron emission tomography, flumazenil

## Abstract

**Aims:**

Increased gamma-aminobutyric acid type A (GABA_A_) receptor activity has been identified in hypersomnolence syndromes, but its role and regional cerebral topography of receptor occupancy in excessive daytime sleepiness (EDS) in Parkinson’s disease (PD) is unknown. Dopaminergic functions or medications may contribute to EDS. Neuroimaging shows evidence of increased brain GABAergic activity in PD. We investigated the relationship between EDS, GABA_A_ receptor binding, striatal dopamine and dopaminergic medication use in PD.

**Methods:**

Six PD patients underwent [^11^C]flumazenil GABA_A_ receptor and [^11^C]dihydrotetrabenazine (DTBZ) dopaminergic positron emission tomography (PET), clinical and Epworth Sleepiness Scale (ESS) assessments.

**Results:**

Mean ESS score was 12.7 ± 4.5. Mean levodopa equivalent dose (LED) was 1,170 ± 616 mg. There was no significant correlation between nigrostriatal [^11^C]DTBZ PET (*R* = 0.138, *p* = 0.792) or disease duration (*R* = 0.209, *p* = 0.902) and ESS scores. In contrast, there was a significant inverse relationship between striatal GABA_A_ receptor activity and ESS scores (β = −0.942, *p* = 0.024), more prominent in the caudate head (β = −0.949, *p* = 0.011). Therefore, frontal cortex binding was explored. A significant inverse relationship between frontal cortical GABA_A_ receptor activity and ESS (*R* = −0.884, *p* = 0.042) was found. A trend toward positive association between LED and ESS (*R* = 0.773, *p* = 0.053), was weakened (β = −0.128, *p* = 0.738) by the addition of caudate head GABA_A_ receptor activity into the model (β = −1.066, *p* = 0.055), suggesting that the association between LED and ESS may be mediated by GABAergic changes in the striatum.

**Conclusion:**

Increased striatal and frontal cortical GABAergic activity is a more significant determinant of daytime somnolence than dopaminergic functions in PD. Findings may augur novel inverse GABA_A_ receptor agonist drug therapy for EDS in PD.

## 1. Introduction

Excessive daytime sleepiness (EDS) is a frequent and disabling non-motor symptom of Parkinson’s disease (PD) that significantly affects patient quality of life^[1]^. Recent epidemiologic studies estimate that 17–40% of individuals with PD experience clinically significant EDS, which is associated with poorer sleep quality, greater depressive symptom burden, impaired attention, increased fall risk, and reduced quality of life^[2–4]^. Moreover, EDS frequently co-occurs with insomnia, rapid eye movement (REM) sleep behavior disorder, and depressive symptoms, contributing to multidimensional deterioration in daytime functioning and psychosocial wellbeing^[5]^.

Although dopaminergic medications contribute to somnolence, EDS is now considered a multifactorial manifestation involving dysregulation across several neurotransmitter systems, including dopaminergic, GABAergic, cholinergic, and glutamatergic pathways^[6,7]^. Gamma-aminobutyric acid (GABA), the major inhibitory neurotransmitter in the central nervous system, acts primarily through gamma-aminobutyric acid type A (GABA_A_) receptors, and alterations in these receptors have been identified in PD using positron emission tomography (PET) and single photon emission computed tomography (SPECT) imaging modalities^[8,9]^. An *in vitro* study confirmed the importance of abnormal GABAergic signaling based on the presence of an endogenous GABA_A_ receptor enhancing compound in the cerebrospinal fluid from patients with primary hypersomnias^[10]^. Furthermore, flumazenil (FMZ), a GABA_A_ receptor benzodiazepine binding site competitive antagonist, normalized vigilance in hypersomnolence studies. Neurophysiological studies further demonstrate that degeneration of nigrostriatal dopaminergic neurons results in increased inhibitory basal ganglia output, with downstream effects on thalamocortical and frontostriatal circuits integral to arousal regulation^[11,12]^. Identifying neural substrates associated with EDS may aid in improving clinical screening, risk stratification, and targeted interventions for sleep–wake dysregulation in PD. Consistent with these neurophysiological observations, *in vivo* imaging studies have demonstrated abnormally increased GABAergic activity in PD, including the striatum. Increased GABA_A_ receptor activity has been recently identified in patients with hypersomnolence syndromes, but its role in daytime sleepiness in PD is unknown^[10]^. To the best of our knowledge, there are no prior GABA_A_ receptor PET or SPECT *in vivo* imaging studies in persons with PD and excessive daytime somnolence. The goal of this study was to examine the relationships between daytime somnolence, cerebral GABA_A_ receptor availability using [^11^C]FMZ PET, nigrostriatal dopaminergic nerve terminals using vesicular monoamine transporter 2 (VMAT2) [^11^C]dihydrotetrabenazine (DTBZ) PET, and dopaminergic medication use in PD. We hypothesized that EDS in PD is better explained by GABAergic changes in the brain than by nigrostriatal nerve terminal integrity or dopaminergic medication.

## 2. Methods

### 2.1 Subjects and clinical test battery

Participants were recruited from the Movement Disorders Clinics at the University of Michigan and Ann Arbor VA Health System. All participants met the U.K. Parkinson’s Disease Society Brain Bank criteria for PD^[13]^. Motor symptom severity was quantified using the motor subsection of the Unified Parkinson’s Disease Rating Scale (UPDRS), assessed in the practically defined “off” state after withholding dopaminergic medications overnight. Cognition was assessed using the Mini-Mental State Exam (MMSE). Daytime sleepiness was measured using the Epworth Sleepiness Scale (ESS), a validated 8-item measure of habitual daytime somnolence^[14]^. The ESS asks subjects to rate how likely they are to doze off or fall asleep during eight typical daily situations (for example, sitting and reading or watching television). Each item is rated on a scale from 0, that would never doze, to 3, which is high chance of dozing. Total scores range from 0 to 24, with higher scores indicating greater daytime sleepiness. ESS ratings were obtained within six months of imaging to ensure temporal proximity to neuroimaging measures. All subjects were on dopaminergic therapy at baseline and withheld their medications overnight before PET scanning to minimize acute pharmacologic effects^[15]^. No subjects were taking benzodiazepines, barbiturates, anticholinergics, or neuroleptic drugs. None had a history of cerebrovascular disease, large-artery stroke, or structural intracranial lesions on screening magnetic resonance imaging (MRI).

The study was approved by the Institutional Review Boards of Ann Arbor Department of Veterans Affairs Medical Center and the University of Michigan. Written informed consent was obtained from all subjects prior to any research procedures.

### 2.2 Imaging techniques

All subjects underwent brain MRI, GABA_A_ receptor benzodiazepine binding site imaging using [^11^C]FMZ PET, and [^11^C]DTBZ VMAT2 PET imaging. [^11^C]FMZ and [^11^C]DTBZ were prepared as described previously^[16–18]^. [^11^C]FMZ binds selectively to BZD-sensitive GABA_A_ receptor subtypes^[8]^, and [^11^C]DTBZ selectively labels VMAT2 to quantify nigrostriatal dopaminergic terminal integrity^[19]^. [^11^C]FMZ dynamic PET imaging was obtained following a bolus injection containing 40% of the total administered 10 mCi dosage over 15 seconds, followed by continuous infusion of the remaining tracer at a constant rate for 62 minutes^[16]^. Similarly, [^11^C]DTBZ dynamic PET imaging was obtained using a bolus injection of 55% of a 15 mCi dose, while the remaining 45% of the dose was continuously infused over the next 60 minutes^[19]^.

MRI was performed on a 3 Tesla Philips Achieva system (Philips, Best, The Netherlands) and PET imaging was performed in 3D imaging mode with an emission computed axial tomography EXACT HR+ tomograph (Siemens Molecular Imaging, Inc., Knoxville, Tennessee) as previously reported^[20]^.

### 2.3 Imaging analysis

PET frames were corrected for motion using rigid-body registration techniques^[21]^. PET images were co-registered to individual MRI scans using mutual-information alignment. Coarse-grained regions of interest (ROIs) were manually and semi-automatically delineated, including striatum, cerebral cortex, thalamus, cerebellar cortex, and pons^[22]^.

FMZ distribution volume ratios (DVRs) were estimated using the Logan plot graphical method^[23]^. The pons served as the reference tissue due to negligible specific [^11^C]FMZ binding^[24,25]^. [^11^C]DTBZ DVRs were similarly estimated using neocortex as reference tissue due to low VMAT2 expression^[19]^. Voxel-wise DVR parametric maps were generated after motion correction and MRI co-registration. All PET-derived measures were visually inspected for quality assurance.

### 2.4 Statistical analysis

To identify potential relevance of disease duration, levodopa equivalent dose (LED), dopaminergic agonist equivalent dose (DAED), disease severity (Movement Disorder Society (MDS)-UPDRS total scores), and age to daytime sleepiness (ESS scores), a set of univariate regression models were fitted predicting ESS scores from each respective covariate. Statistically significant or trending (*p* < 0.1) covariates were used to inform subsequent regression analyses.

Associations between [^11^C]FMZ DVR values and ESS scores were analyzed using regression models. [^11^C]FMZ DVR of coarse-grained ROIs (excluding the pons) were entered into forward stepwise selection procedure, with striatal [^11^C]DTBZ DVR values included as a fixed covariate to account for dopaminergic denervation. An additional analysis was performed, predicting ESS scores from the selected coarse-grained volume(s) of interest after adjustment for their respective [^11^C]FMZ K1 proxy flow rates, to confirm the biological specificity of observed correlation.

A secondary forward stepwise selection procedure was separately performed on finer sub-region(s) of the coarse-grained ROI(s) entered into the primary regression model. The [^11^C]FMZ DVR of selected sub-regions were entered into a regression model predicting ESS scores, with relevant clinical covariates (duration or LED) adjusted for in the model. Normality of model residuals was assessed for all linear regression models using the Shapiro-Wilk test. Analyses were conducted using SAS version 9.3, with α < 0.05 considered statistically significant.

## 3. Results

This cross-sectional study included 6 subjects with PD (5 males, 1 female; mean age 65.5 ± 4.6 years) with a mean disease duration of 7.5 ± 1.4 years and a mean LED of 1,170 ± 616 mg. Mean Hoehn and Yahr stage^[26]^ was 2.75 ± 0.27, and mean MMSE score was 27.3 ± 2.9. Total MDS-UPDRS scores along with Part I, II, and III sub-scores are presented in Table 1.

Mean ESS score was 12.7 ± 4.5 (range 7–19). The univariate correlation between ESS scores and both disease duration (*R* = 0.209, *p* = 0.902), disease severity (*R* = 0.442, *p* = 0.381), and age (*R* = 0.361, *p* = 0.482) was not statistically significant. The univariate correlation between ESS scores and LED demonstrated a non-significant trend for a positive association (*R* = 0.773, *p* = 0.053), whereas the correlation between ESS scores and DAED did not approach statistical significance (*R* = 0.118, *p* = 0.823). There was no significant correlation between ESS scores and striatal dopaminergic terminal integrity measured with [^11^C]DTBZ (*R* = 0.138, *p* = 0.792).

**Figure 1.**
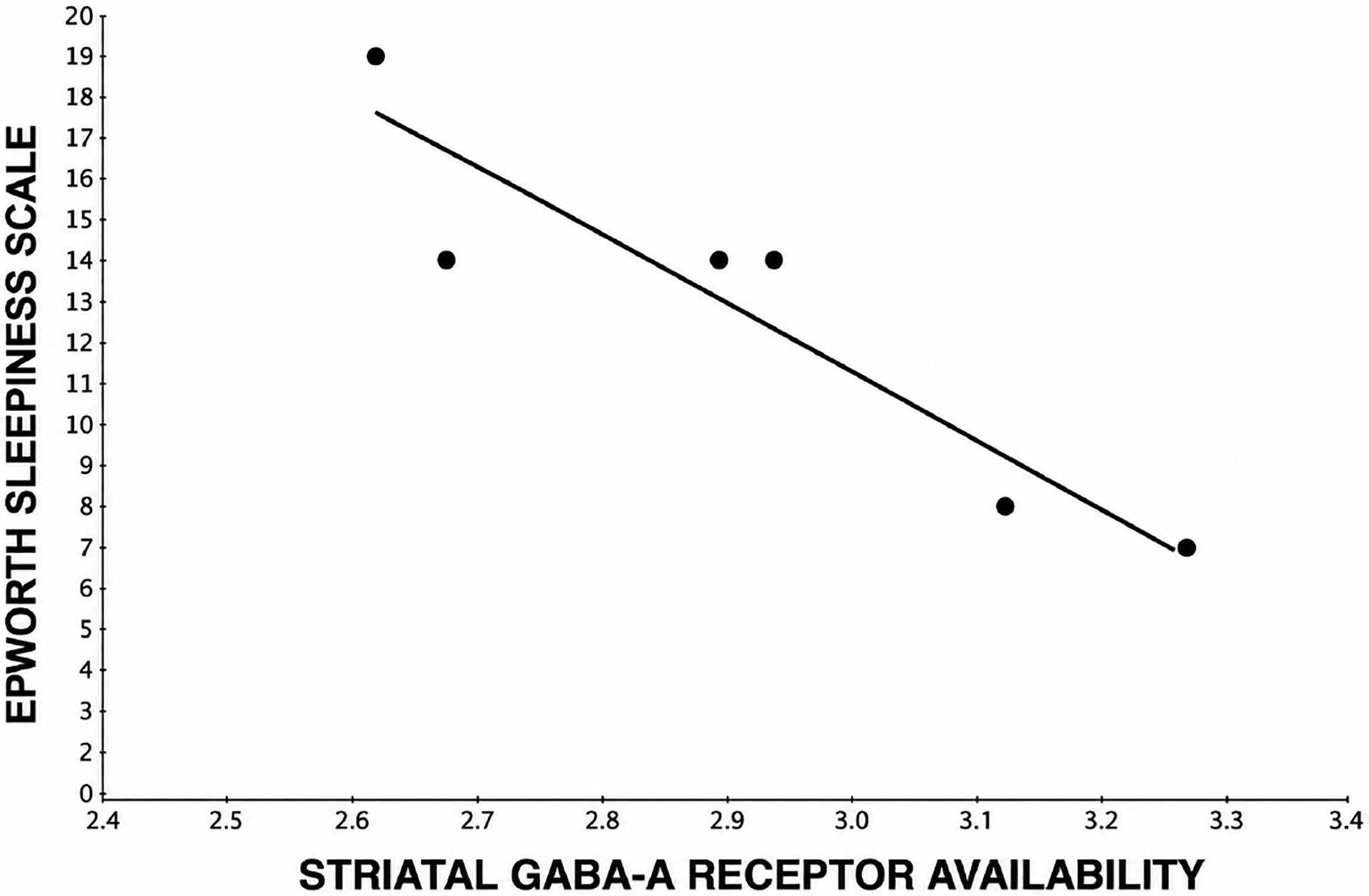
Lower availability of striatal GABA_A_ receptor BZD binding sites (reflecting hyper-GABAergic state) associates with higher ESS scores (β = −0.942, *p* = 0.024), demonstrating a correlation with excessive DTS in PD. GABA_A_: gamma-aminobutyric acid type A; BZD: benzodiazepine; ESS: Epworth sleepiness scale; DTS: daytime sleepiness; PD: Parkinson’s disease.

GABA_A_ receptor availability measured using [^11^C]FMZ PET only in the striatum was selected for by the coarse-grained forward selection procedure, and demonstrated a robust, significant inverse association with ESS scores (β = −0.942, *p* = 0.024) independently of striatal dopaminergic integrity (β = −0.074, *p* = 0.758) as shown in Figure 1. Association between striatal GABA_A_ receptor availability and ESS was also preserved (β = −0.862, *p* = 0.03) after adjustment for striatal [^11^C]FMZ K1 proxy flow, which itself was not a significant predictor (β = −0.158, *p* = 0.528), indicating that the observed associations reflected receptor-specific effects rather than differences in perfusion or tracer delivery.

A fine-grained forward-selection was therefore performed predicting ESS scores from [^11^C]FMZ PET uptake in striatal sub-regions, including ventral/dorsal/anterior/posterior putamen and inferior/middle/head of caudate, with striatal dopaminergic integrity as a fixed covariate. The caudate head was selected as the best sub-regional [^11^C]FMZ PET uptake predictor of ESS scores independently of striatal dopaminergic innervation (β = −0.949, *p* = 0.011). Because of the strong association found in the caudate nucleus, we also explored GABA_A_ receptor availability in the frontal cortical projection area and found a significant inverse association with ESS scores (*R* = −0.884, *p* = 0.042).

**Figure 2.**
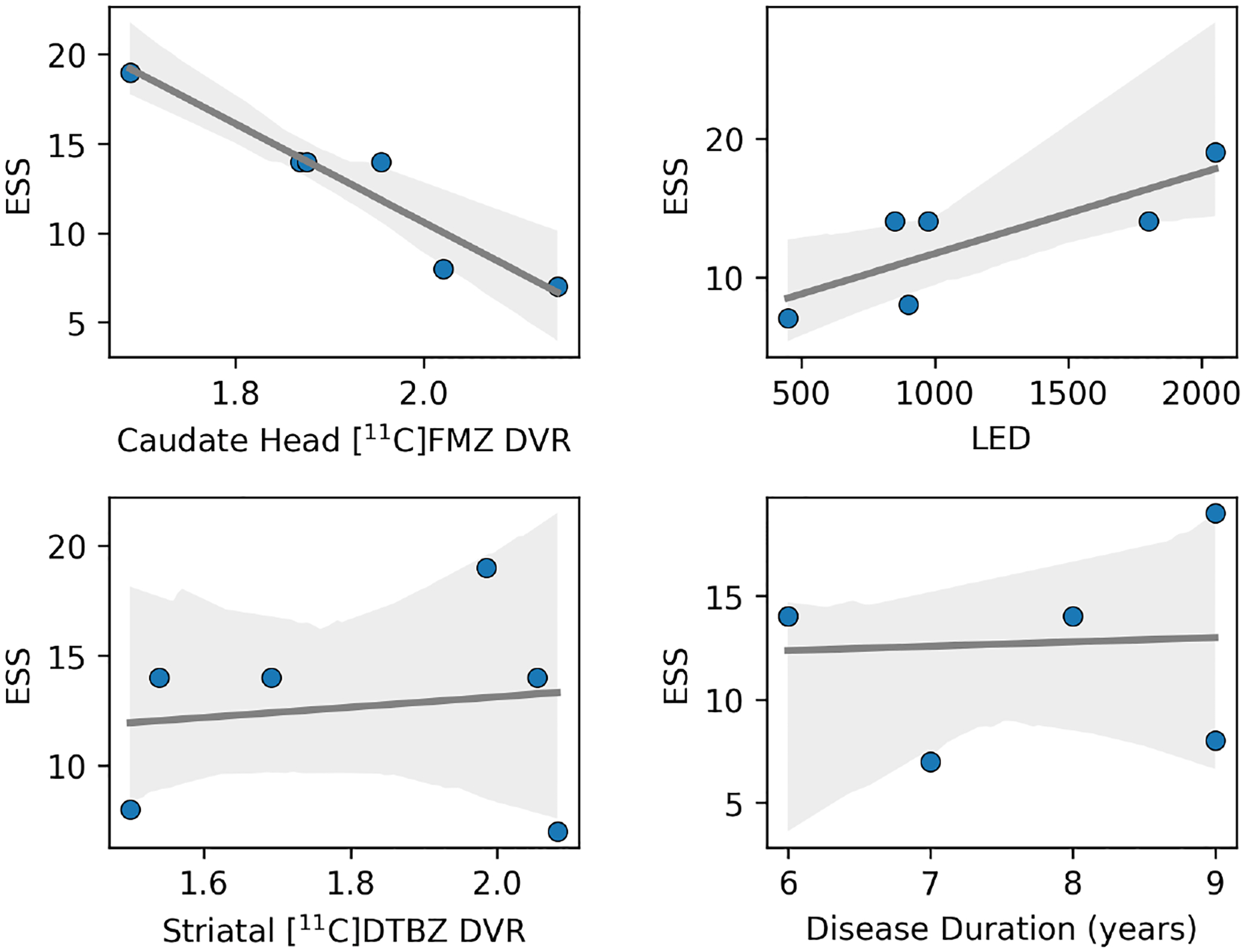
ESS was most strongly correlated with caudate head GABA_A_ receptor availability ([^11^C]FMZ DVR) and LED. Striatal dopaminergic integrity ([^11^C]DTBZ DVR) and disease duration did not substantially correlate with ESS. ESS: Epworth sleepiness scale; GABA_A_: gamma-aminobutyric acid type A; LED: levodopa equivalent dose; FMZ: flumazenil; DVR: distribution volume ratio; DTBZ: dihydrotetrabenazine.

The addition of caudate head [^11^C]FMZ PET uptake into the model predicting ESS scores from LED substantially weakens the correlation between ESS scores and LED on the preliminary univariate analysis (β = −0.128, *p* = 0.738), while caudate head [^11^C]FMZ PET uptake remains a strong predictor of ESS scores (β = −1.066, *p* = 0.055), suggesting that the association between LED and ESS scores may not be independent of caudate head GABA_A_ receptor availability. Given these findings, a post-hoc analysis was performed to identify whether any univariate correlation might be present between caudate head [^11^C]FMZ PET uptake and LED. A statistically significant inverse univariate correlation was observed (*R* = −0.875, *p* = 0.022), suggesting that higher LED may associate with greater daytime sleepiness indirectly through its negative association with caudate head GABA_A_ receptor availability. A selection of relevant univariate correlations are presented in Figure 2.

## 4. Discussion

EDS represents a clinically relevant and often underrecognized non-motor disease burden in PD. While EDS is often linked to dopaminergic functions, particularly dopaminergic medication use and the use of GABAergic drugs like benzodiazepines, the relationship between daytime sleepiness and PD-related GABAergic hyperactivity and its topography remains poorly understood. We explored this relationship using [^11^C]FMZ to quantify GABA_A_ receptor availability as a measure of GABAergic activity and the ESS to measure EDS burden. We found strong inverse associations between EDS and GABA_A_ receptor benzodiazepine-site availability in striatal and frontal cortical regions in PD. These findings suggest that GABAergic inhibitory regulation within frontostriatal circuits may contribute to arousal disturbances in PD. Notably, the univariate correlation between LED and EDS was substantially weakened by the presence of caudate head GABA_A_ receptor availability in the model, suggesting that the previously observed association between dopaminergic replacement therapy and EDS^[27]^ may be mediated by changes in the striatal GABAergic system. Furthermore, we did not find a direct association between EDS and nigrostriatal dopaminergic activity, as determined by [^11^C]DTBZ PET. Striatal [^11^C]FMZ K1 radioligand flow delivery rates did not correlate with ESS, indicating that the observed associations reflected receptor-specific effects rather than differences in perfusion or tracer delivery. This is consistent with prior molecular imaging evidence showing that cortical and striatal GABA_A_ receptor changes in PD are not explained solely by perfusion or synaptic density differences^[1,9]^.

Increased inhibitory basal ganglia output following dopamine loss has long been established^[11,12]^. GABA co-release from nigrostriatal dopaminergic terminals is a well-established phenomenon where dopamine neurons release both dopamine and GABA. This co-release occurs despite the absence of the canonical vesicular GABA transporter, which is normally required for GABA packaging into synaptic vesicles^[28,29]^. Instead, GABA is taken up into vesicles via **GABA transporter-1** from the extracellular space and packaged into synaptic vesicles using **VMAT2**, the same transporter responsible for loading dopamine into vesicles^[28]^. This unique mechanism allows GABA and dopamine to be co-packaged and co-released from the same vesicles. In this respect, GABA is not just a co-transmitter but a key modulator of dopaminergic signaling, fine-tuning both the timing and magnitude of dopamine release in the striatum. However, we did not find any specific associations with nigrostriatal VMAT2 PET binding.

In **PD**, degeneration of dopaminergic neurons leads to altered activity in the striatal direct and indirect pathways^[30]^. The striatum sends GABAergic efferents primarily to the **globus pallidus** and **substantia nigra**, forming part of the basal ganglia-thalamo-cortical loop. This results in **increased GABAergic output** from the basal ganglia to the thalamus and contributes to reduced thalamocortical drive and **hypokinetic motor symptoms**^[31]^. The caudate nucleus projects to the frontal cortex via a thalamocortical loop forming part of the corticobasal ganglia-thalamocortical circuit^[32]^ that may be related to the EDS in PD.

There may also be an independent role of altered GABAergic activity in the frontal cortex and EDS in PD. Prior [^11^C]FMZ PET imaging studies have shown evidence of reduced GABA_A_ receptor binding in the frontal cortex of early PD patients, correlating with cognitive deficits and frontal lobe dysfunction^[33]^. For example, increased GABAergic tone in arousal-regulation regions like the prefrontal cortex has been implicated in narcolepsy^[34]^. Our findings suggest that striatal and frontal GABA_A_ receptors may modulate sleepiness in PD via diverse neural network dysregulations^[35]^.

Aging is one of the biggest known risk factors for the development of PD^[36]^. Disruptive processes that underpin neurodegeneration of nigrostriatal dopaminergic projections in PD, including oxidative stress and metabolic dysregulation, reach their biological threshold in PD^[37]^. The overlap between aging and PD related brain changes is further supported by incidental Lewy body pathology, which is observed in > 20% of older adults who died prior to developing PD^[38]^. Given accumulating evidence for overlap in mechanism between PD and age-related brain pathology, changes in striatal and frontal cortical GABA_A_ receptor availability may underpin daytime sleepiness also in older adults. The GABAergic system has been previously shown to be implicated in patients with sleep disorders, both with^[35]^ and without PD^[39]^. Interventions targeting sleep quality through the GABAergic system have also shown promise^[40,41]^. Though further studies in older adults without PD are needed to provide supportive evidence, the findings of the present work provide a promising future direction for investigating daytime sleepiness in older adults, which may pave the way for new treatment approaches in geriatric medicine.

A limitation of the study is the small sample size. However, the linear nature of the correlations between striatal and frontal cortical findings and EDS points to robustness of the observations, although further research is needed for validation. Positive findings may augur the use of flumazenil-based therapies in the treatment of EDS in PD.

## 5. Conclusion

Our findings indicate that increased striatal and frontal cortical GABAergic activity is a more significant determinant of EDS than dopaminergic functions alone in PD. These results support a potential inhibitory mechanism underlying EDS in PD. Additional studies with larger samples are needed to further elucidate the potential mediation between LED and EDS via striatal GABA_A_ receptor availability.

## Figures and Tables

**Table 1.** MDS-UPDRS scores by patient.

Patient	Total Score	Part I	Part II	Part III
1	65	8	8	49
2	71	17	9	45
3	48	16	4	28
4	37	7	8	22
5	87	16	10	61
6	45	5	3	37

MDS: Movement Disorder Society; UPDRS: unified Parkinson’s disease rating scale.

## Data Availability

The data and materials could be obtained from the corresponding author.
